# Effect of dental implant surface roughness in patients with a history of periodontal disease: a systematic review and meta-analysis

**DOI:** 10.1186/s40729-019-0156-8

**Published:** 2019-02-13

**Authors:** Anton Dank, Irene H. A. Aartman, Daniël Wismeijer, Ali Tahmaseb

**Affiliations:** 1 Section of Oral Implantology and Prosthetic Dentistry, Academic Centre for Dentistry Amsterdam (ACTA), University of Amsterdam and Vrije Universiteit Amsterdam, Gustav Mahlerlaan 3004, 1081 LA Amsterdam, The Netherlands; 2 Department of Social Dentistry, Academic Centre for Dentistry Amsterdam (ACTA) University of Amsterdam and Vrije Universiteit Amsterdam, Gustav Mahlerlaan 3004, 1081 LA Amsterdam, The Netherlands

**Keywords:** Dental implants, Implant surface, Periodontal, Bone loss

## Abstract

**Background:**

To review the literature on the effect of dental implant surface roughness in patients with a history of periodontal disease. The present review addresses the following focus question: Is there a difference for implant survival, mean marginal bone loss, and the incidence of bleeding on probing in periodontally compromised patients receiving a machined dental implant or rough surface dental implant?

**Methods:**

Electronic and manual literature searches were conducted on PubMed/MEDLINE and the Cochrane Library on studies published until May 2018 to collect information about the effect of machined, moderately rough, and rough dental implant surfaces in patients with a history of periodontal disease. The outcome variables implant survival, mean marginal bone level, and the incidence of peri-implantitis and bleeding on probing were evaluated. Meta-analysis was performed to obtain an accurate estimation of the overall, cumulative results.

**Results:**

Out of 2411 articles, six studies were included in this systematic review*.* The meta-analysis of the implant survival and implant mean marginal bone loss revealed a risk ratio of 2.92 (CI 95% 0.45, 18.86) for implant failure and a total mean difference of − 0.09 (CI 95% − 0.31, 0.14) for implant mean marginal bone loss measured in a total group of 215 implants, both not statistically significant.

**Conclusions:**

Due to lack of long-term data (> 5 years), the heterogeneity and variability in study designs and lack of reporting on confounding factors, definitive conclusions on differences in implant survival, and mean marginal bone loss between machined and moderate rough implants in periodontally compromised patients cannot be drawn. Future well-designed long-term randomized controlled trials are necessary to reveal that machined surfaces are superior to moderately rough and rough surfaces in patients with a history of periodontal disease.

**Electronic supplementary material:**

The online version of this article (10.1186/s40729-019-0156-8) contains supplementary material, which is available to authorized users.

## Background

Rough titanium implants are currently the standard treatment in implant dentistry [1]. They are roughly divided into three different types of surface roughness (*S*_a_): machined/minimal (± 0.5 μm), moderate (1.0–2.0 μm), and rough (> 2.0 μm) [2]. Generally, rougher implant surfaces have greater bone-to-implant contact [3]. In a randomized controlled clinical trial, it has been demonstrated that higher initial clinical survival rates are achieved when implants with a moderate rough surface are used, compared with machined implants [4]. Moreover, greater forces are required for rougher surfaced implants to be removed [5].

On the other hand, a disadvantage of this increased roughness might be that it facilitates bacterial adhesion at the implant surface [6]. Once they become exposed, rough surface implants are more vulnerable to attract plaque. This disadvantage may entail that in some patients inflammation around rougher-surfaced implants might occur easier. Some patient groups are more vulnerable for this than others, e.g., periodontally compromised patients and smokers. They tend to have a higher risk of implant failure [7–16]. Clinicians are commonly placing dental implants with various surface roughness and modifications including plasma-sprayed, acid-etched, blasted, oxidized, hydroxyapatite-coated, or combinations of these procedures in these risk groups [17, 18].

In their systematic review, Saffi et al. have stated that periodontally compromised patients were significantly at higher risk of implant failure and increased marginal bone loss compared with periodontally healthy patients [19]. The microbiotic flora involved in peri-implant disease, i.e., peri-implantitis, resembles the flora associated with periodontitis [20–22]. Teeth might act as a reservoir for the colonization of the peri-implant sulcus. Within 2 weeks after one-stage implant placement or abutment connection in a partially edentulous patient, the peri-implant sulcus becomes colonized with bacteria similar to the neighboring natural teeth [23]. However, several other studies have reported no association between failing implants and history of periodontal disease [17, 24, 25]. Another systematic review on implant treatment in periodontally compromised patients has demonstrated high survival rates of implants in individuals with a history of periodontitis-associated tooth loss [26].

Several animal studies have suggested that the roughness of the implant surface influences the progression of peri-implantitis and the outcome of peri-implantitis treatment [27–31]. There is some evidence in men showing that machined implants are less prone to peri-implantitis compared with implants with rougher surfaces [32]. Moreover, implants with a rough surface have higher rates of late implant failures compared with machined or moderately rough implants [1, 33]. Concerning the peri-implantitis treatment, Esposito et al. have shown in a systematic review that the progressive marginal bone loss around rough implants may be more difficult to halt than around machined implants [34].

Therefore, the question arises as to whether periodontally compromised patients might benefit from placing machined implants, in spite of their relatively higher early failure rate [35]. Thus, the aim of this systematic review is to evaluate the effect of different implant surface roughness on implant survival rate, mean marginal bone loss, and the incidence of peri-implantitis in periodontally compromised patients. For this reason, the present review addresses the following PICO (patient-intervention-comparison-outcomes) question: Is there a difference for implant survival, mean marginal bone loss, and the incidence of bleeding on probing (O) in periodontally compromised patients (P) receiving a machined dental implant (I) or rough surface dental implant (C)? Preferably, this question is answered in randomized controlled trials.

## Methods

This study followed the PRISMA statement guidelines and is registered at PROSPERO under registration code CRD42018093063. A review protocol does not exist.

### Search strategy

The listed PICO question is used in the present systematic search strategy. The electronic data resources consulted were PubMed/MEDLINE and Cochrane Library, including all published clinical studies until May 2018. The results were limited to studies written in English.

The following terms were imported in the search strategy on PubMed/MEDLINE: dental AND (implant OR implants OR implantation OR implantology) AND (surface OR surfaces) AND (periodontics OR periodontology OR periodontal disease). The following terms were used in the search strategy on the Cochrane Library: ((((dental AND (implant) OR implants) OR implantation) OR implantology)) AND ((((surface) OR surfaces) OR surface topography)) AND (((periodontics) OR periodontology) OR periodontal disease).

### Inclusion and exclusion criteria

Eligibility criteria included human randomized and non-randomized controlled trials and prospective and retrospective cohort studies. All periodontally compromised patients studied should be successfully periodontally treated and enrolled in a high-quality maintenance care program after completion of active treatment. At least ten patients had to be examined. Only studies with a follow-up of at least 3 years were included. Studies with orthodontic implants, immediate placed and/or loaded implants, and implants placed in combination with sinus floor elevation were excluded. Also, case reports, technical reports, animal studies, cadaver studies, in vitro studies, and review papers were rejected.

### Study selection

Two reviewers (A.D. and A.T.) screened all identified titles and abstracts independently. In addition, the reference lists of the subsequently selected abstracts and the bibliographies of the systematic reviews were searched manually. For studies appearing to meet the inclusion criteria, or for which insufficient data in the title and abstract was available, the full text was obtained. Disagreements were solved through discussion between the authors. Finally, the full-text evaluation of the remaining publications was done using the above-listed inclusion and exclusion criteria.

### Data extraction and meta-analysis

Two reviewers independently extracted data from the included studies. Disagreements were again resolved through discussion. Corresponding authors were contacted when data were incomplete or unclear. With respect to the listed PICO question, data were sought for (P) periodontally compromised and patients without a history of periodontitis receiving dental implant placement, (I) machined surface dental implants, and (C) rough surface dental implants. Both reviewers evaluated the following primary outcomes (O): implant survival rate after 3–10 years and implant mean marginal bone loss. Implant mean attachment loss, incidence of peri-implantitis, and incidence of bleeding on probing around implants were evaluated as secondary outcomes. Meta-analysis was attempted for studies reporting the same outcome measures. Finally, funding sources of the selected studies have been checked.

### Quality of the studies

Quality assessment of the selected studies was executed by using the Cochrane Collaboration Tool (http://ohg.cochrane.org) for randomized controlled trials (RCTs) including random sequence generation, allocation concealment, blinding of participants, incomplete outcome data, selective reporting, and other bias. The Newcastle-Ottawa scale (http://www.ohri.ca/programs/clinical_epidemiology/oxford.asp) was applied for non-randomized studies to judge each included study on selection of studies, comparability of cohorts, and the ascertainment of either the exposure or outcome of interest. Stars were awarded such that the highest quality studies were awarded up to nine stars.

### Statistical analysis

Review Manager 5.0 was used for statistical analysis. Differences in means and risk ratios were used as principal summary measures. Forest plots were created to visualize the differences between groups.

## Results

**Fig. 1:**
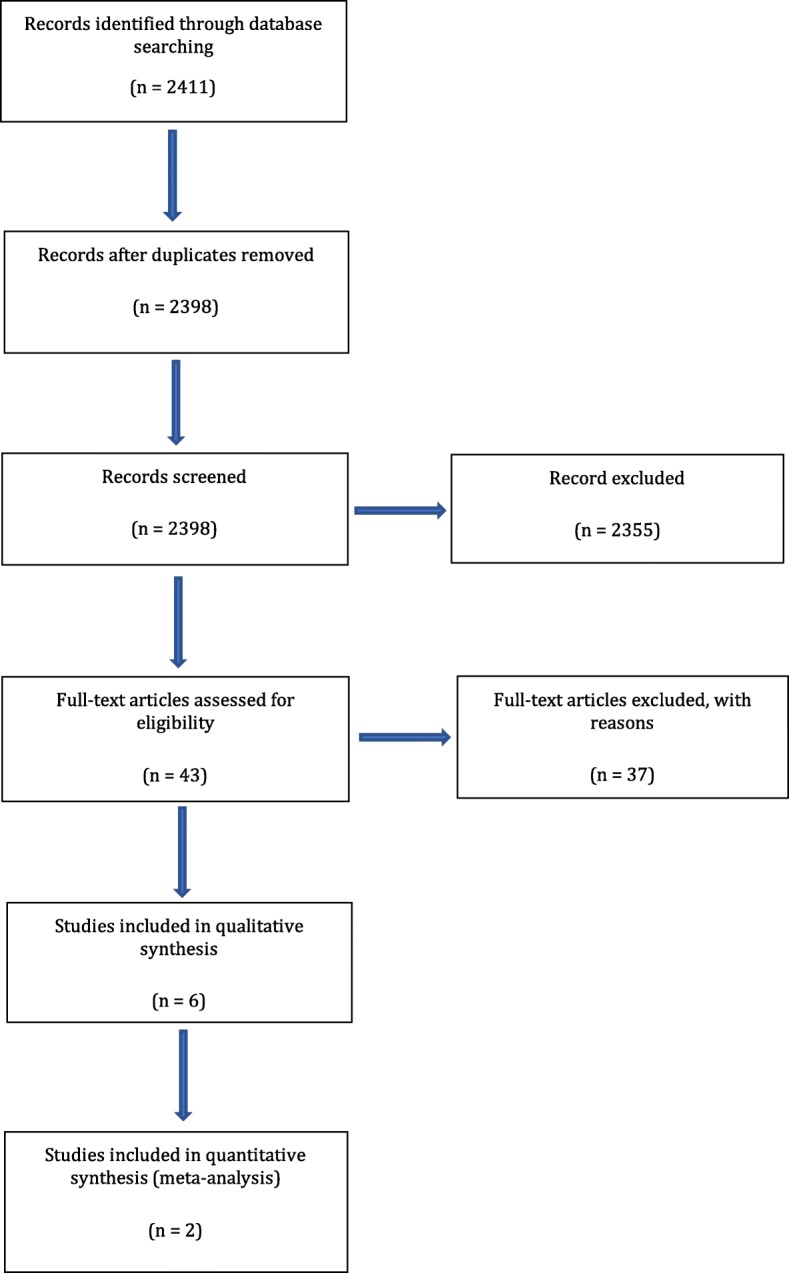
Flowchart of the search strategy

The initial electronic database search on PubMed/MEDLINE and Cochrane library resulted in 2411 titles. Thirteen articles were cited in both databases (duplicates). After screening the abstracts, 45 relevant titles were selected by two independent reviewers and 2353 were excluded for not being related to the topic. Following examination and discussion by the reviewers, 43 articles were selected for full-text evaluation. Hand searching of the reference lists of the selected studies did not deliver additional papers. After pre-screening, application of the inclusion and exclusion criteria and handling of the PICO questions, six studies remained (ten studies did not report on a periodontally compromised group, two studies did not on a periodontally healthy group, one paper reported only on immediate loading, one paper only on immediate restoration, two studies only inform on micro-implants, two were microbiological studies, in eleven studies the periodontal status was not mentioned, five studies only reported on moderate rough implants, one only on machined surface implants, one paper did not report on primary outcome mean marginal bone loss, and from one paper only the article with 5-year results was included, while the corresponding article with 20-year results was excluded) [36–41]. They were used for data extraction and statistical analysis. Of the six included studies, two were RCTs [38, 40]. Figure 1 illustrates a flowchart of the search results.

### Study characteristics

Within the remaining group of six included studies in one study, 5.3% of the patients have controlled diabetes mellitus (Gallego et al.) [41]. With this exception, all patients were generally and periodontally healthy at the moment of implantation (Table 1). Four out of the six studies looked only at periodontally compromised patients (Sayardoust et al., Wennström et al., Nicu et al., and Gallego et al.), whereas the other two looked both at periodontally healthy patients and periodontally compromised patients (Aglietta et al. and Matarasso et al.) [36–41]. One study contained only smokers (Aglietta et al.), one study contained only non-smokers (Matarasso et al.), another study made separate groups for smokers and non-smokers (Sayardoust et al.), and three studies mixed both smokers and non-smokers (Wennström et al., Nicu et al., and Gallego et al.) [36–41]. Different periodontal diagnoses were reported within the periodontally compromised patient group: mild, chronic, moderate, and advanced. Two studies did not report on the use of antibiotics (Aglietta et al., Matarasso et al.) [36, 39]. In one study, no antibiotics were used (Sayardoust et al.) [37]. In the study of Nicu and coworkers, post-operative antibiotics were prescribed: 3 × 500 mg amoxicillin, 5 days [40]. In the study of Wennström and coworkers, the patient received 2 g of penicillin 1 h pre-operatively and 2 × 1 g penicillin, 7 days post-operatively [38]. In the study protocol of Gallego and coworkers, 3 × 500 mg amoxicillin for 7 days was prescribed [41].

**Table 1:** Study characteristics and individual results included studies

Study (first author and year of publication)															
	Design	General health	Perio health	Perio status	Perio diagnosis	Smoking status	Smokers (%)	Perio recall *x*/year	Follow-up (months)	Follow-up range	No. of patients	Mean age	Dental status	Implants surface	No. of implants
Aglietta, 2010	Retro	Yes	Yes	PHPHPCPC	Chronic	SSSS	100	Regular	120	ND	10 10 10 10	51.2 51.5 51.3 51.7	Partial	MSRSMSRS	10 10 10 10
Sayardoust, 2013	Retro	Yes	Yes	PCPCPCPC	Advanced	NSNSSSSS	50	Regular	60	ND	20 20 20 20	59.8 63.2 54.2 53.5	Partial and full	MSRSMSRS	66 52 78 56
Wennström, 2004	Pros	Yes	Yes	PCPC	Moderate-to-advanced chronic	S and NS	33	2–3/years	60	ND	51	59.5	Partial	MSRS	67 70
Matarasso, 2010	Retro	Yes	Yes	PCPCPHPH	Chronic	NSNSNSNS	O	Regular	120	ND	20 20 20 20	47.2 46.5 47.5 48.1	Partial	MSRSMSRS	20 20 20 20
Nicu, 2012	Pros	Yes	Yes	PCPC	Moderate to severe chronic	S and NS	44	Regular	36	ND	1818	55.4 55.4	Partial and full	MSRS	39 39
Gallego, 2018	Retro	5.3% controlled diabetes	Yes	PCPCPC	Mild to Advanced	S and NS	28	3–4/years	36	ND	27 74 167	61. 0 61.2 61.1	Partial	MSHSRS	72 14 55 38
	Site of placement1. Max and/or mand2. Ant and/or post	Stage	AB	Early implant loss (%)	Late implant loss (%)	Implant survival (%)	Implant mean marginal bone loss	Implant mean marginal bone loss range	Implant mean attach level loss	Implant mean attachment level Loss range	BoP (%)	Peri-implantitis (%)	Type of prosthesis	Screw-retained or Cemented	Implant surface
Aglietta, 2010	1. Max and mand2. Ant and post	2121	NR	0.0 0.0 0.0 0.0	10.0 0.0 10.0 20.0	90.0 100 90.0 80.0	2.65 2.51 3.47 3.77	0.31 0.31 1.09 1.43	ND	ND	18.1 20.1 19.3 18.7	ND	Single unit	NR	Turned, TPS surface
Sayardoust, 2013	1. Max and mand2. NR	2	No	2.5 3.1 12.1 3.0	0.6 0.8 3.0 0.8	96.9 96.1 84.9 96.2	0.84 1.26 1.54 1.16	0.14 0.15 0.21 0.15	ND	ND	< 15%	ND	Single unit, FPD	Screw	Turned, oxidized surface
Wennström, 2004	1. Max and mand2. Ant and post	2	Yes	1.45 0.0	1.45 9.3	97.1 98.6	0.33 0.48	1.07 0.95	ND	ND	5.0	4.7	FPD, extension FPS’s	Screw	Turned, TiO2-blasted surface
Matarasso, 2010	1. Max and mand2. Ant and post	2121	NR	0.0 0.0 0.0 0.0	5.0 15.0 5.0 5.0	95.0 85.0 95.0 95.0	2.78 2.32 1.95 1.43	0.48 0.41 0.42 0.38	ND	ND	ND	ND	Single unit	NR	Turned, TPS
Nicu, 2012	1. Max and mand2. Ant and post	2	Yes	4.2 0.0	0.0 0.0	95.8 100	0.98 1.02	0.63 0.72	3.44.0	1.41.9	59 69	ND	FPD, overdenture	Screw	Turned, TiUnite
Gallego, 2018	1. Mand2. Post	1	Yes	0.0 0.0 0.0	0.0 0.0 0.0	100 100 100	0.96 0.77 1.48	0.49 0. 60 1.09	ND	ND	9.6 13.5 13.0	ND	FPD	Screw	Turned, osseotite, TiUnite

There is some variation in the follow-up between the different studies. Two studies had a follow-up of 5 years (Sayardoust et al. and Wennström et al.), two had a follow-up of 10 years (Aglietta et al. and Matarasso et al.), and for two studies, it was 3 years (Nicu et al. and Gallego et al.) [36–41]. All included periodontally compromised patients participated in a regular periodontal maintenance program. The mean age in the six included studies containing 555 patients ranged from 46.5 to 63.2 years. Both partially and fully edentulous patients were included, and implants were placed in both the maxilla and the mandible in five studies [36–40]. In one study (Gallego et al.), implants were exclusively placed in the posterior mandible [41]. In three studies, all implants were placed two-staged (Sayardoust et al., Wennström et al., and Nicu et al.), and in two studies, only machined implants were placed two-staged (Aglietta et al. and Matarasso et al.) [36–40].

### Results of the individual studies

As measured in the six included studies containing 1342 implants, implant survival rates for machined surface implants ranged from 84.9 to 97.1%, while for rough surface implants ranged from 80 to 100% (Table 1). Machined surface implants display a range from 0.33 (CI 95% − 0.74, 1.40) to 3.47 (CI 95% 2.38, 5.46) for implants mean marginal bone loss, while rough surface implants display a range from 0.48 (CI 95% − 0.47, 1.43) to 3.77 (CI 95% 2.34, 5.20). Bleeding on probing varies from 5.0 to 69% [36–41].

### Quality of the studies

**Fig. 2:**
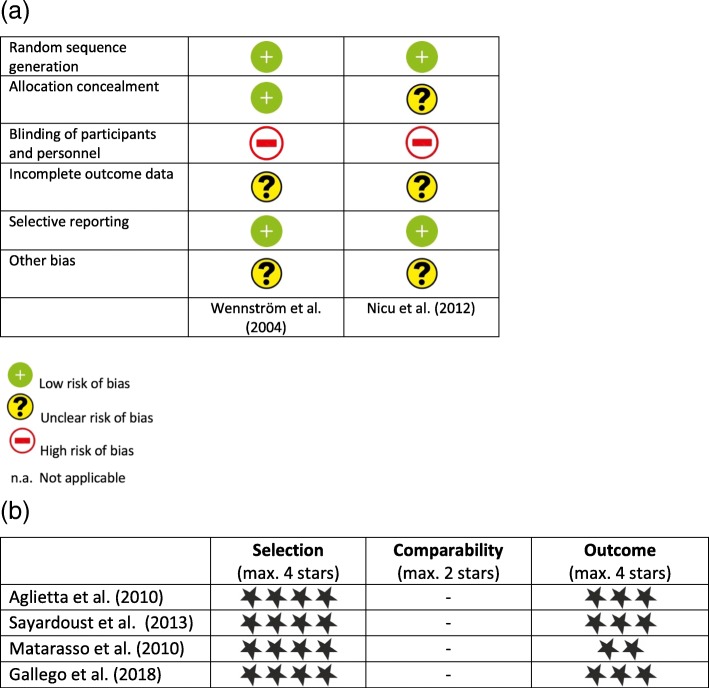
**a** Presentation of risk of bias evaluation for included RCTs according to the Cochrane Collaboration’s tool. **b** Presentation of risk of bias evaluation for included non-RCTs according to the Newcastle-Ottawa assessment scale

Quality assessment of the included prospective studies was executed according to the Newcastle-Ottawa scale. The two studies were of moderate quality, and risk of bias is present in both [38, 40]. In Wennström et al., we accounted several losses to follow-up: three patients had died and one patient had discontinued therapy [38]. Each patient received a minimum of two implants, and by randomization, every second implant inserted had a machined surface and the remaining had a rough surface. The absence of a split-mouth design creates a risk of bias because the evaluated groups are not completely comparable. The authors accomplish concealment of allocation by proper blinding using a randomization code which was made available after the surgeon had made his osteotomies. Clinical scores were assessed by an examiner not involved in the trial. In our validity assessment, we found that the Nicu et al. paper is at high risk of bias [40]. The extra groove in the TiUnite implants precluded proper blinding, although both implant types had the same macro design. In every patient, two or more machined surface implants and two or more rough surface implants were randomly selected by a computer randomization program. Figure 2a, b displays assessment of the risk of bias for included RCT and non-RCT studies.

### Synthesis of results

**Fig. 3:**
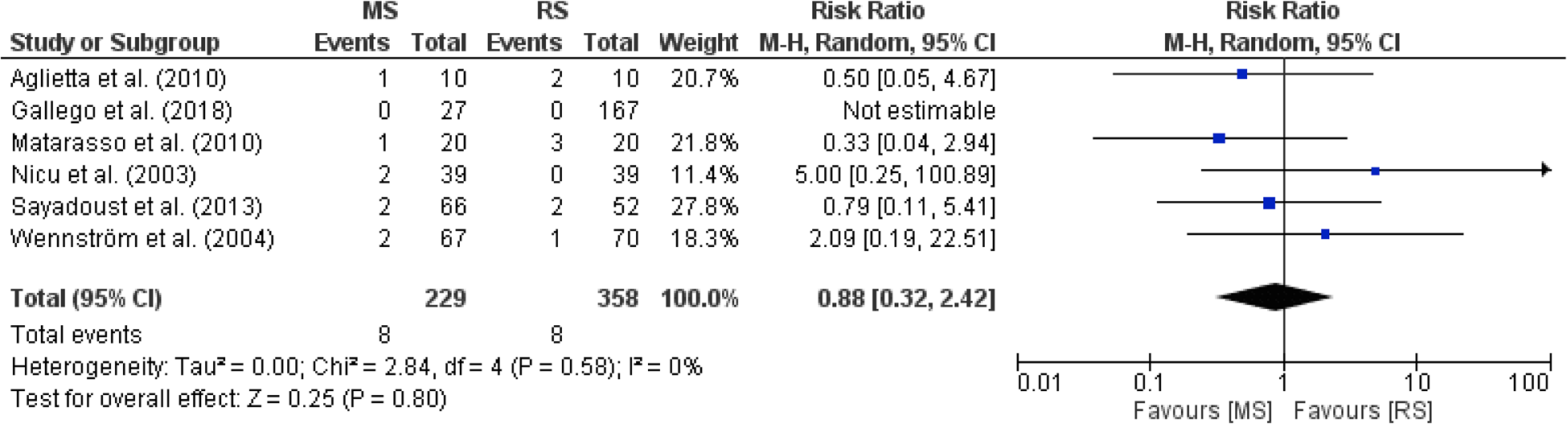
Forest plot on differences in implant survival between MS and RS groups in all included studies

**Fig. 4:**
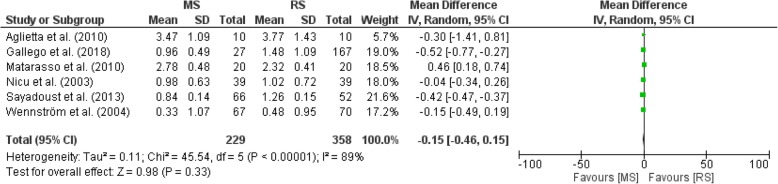
Forest plot on differences in implant mean marginal bone loss between MS and RS groups in all included studies

**Fig. 5:**
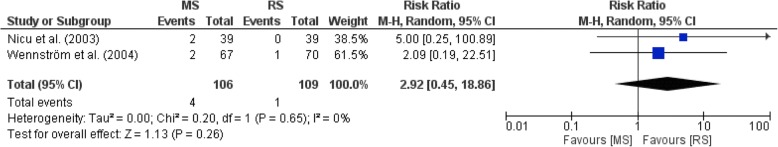
Forest plot on differences in implant survival between MS and RS groups in all included RCT's

**Fig. 6:**
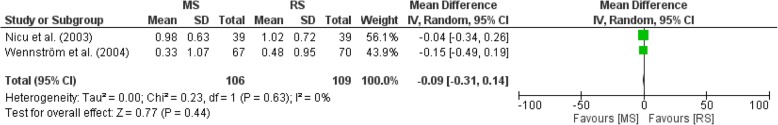
Forest plot on differences in implant mean marginal bone loss between MS and RS groups in alle included RCT's

Figure 3 illustrates a forest plot showing no significant differences in implant survival between MS and RS groups in all included studies [36–41]. The implant mean marginal bone loss in the remaining group of six included studies containing 1342 implants ranged from 0.33 to 3.77 mm, with a minimum and maximum of − 0.74 and 5.20 mm, respectively [36–41]. The forest plot in Fig. 4 demonstrates no significant differences in implant mean marginal bone loss between MS and RS groups in all included studies [36–41]. One study (Matarasso et al.) did not report on bleeding on probing (BoP), whereas four studies reported BoP varying from 5.0 to 69.0% (Aglietta et al., Sayardoust et al., Wennström et al., Nicu et al., and Gallego et al.) [36–41]. Meta-analysis was in addition separately performed on the two included RCTs [38.40]. Figure 5 illustrates a forest plot showing no significant differences (*P* > .05) in implant survival between MS and RS groups in both studies [38, 40]. Figure 6 shows no significant differences (*P* > .05) in implant mean marginal bone loss between MS and RS groups in both RCTs [38, 40]. A limitation of the performed meta-analysis is that the merge of periodontally compromised smokers and non-smokers could not be avoided.

## Discussion

The current study reviews the literature on the effect of dental implant surfaces in patients with a history of periodontal disease. The six included papers comprised both retrospective and prospective studies [36–41]. The two prospective randomized clinical trials were analyzed separately [38, 40]. As demonstrated by equality of the risk ratios and on account of the limited amount of included studies, we could not find any difference between machined surface implants and rough surface implants in both implant survival rate and implant mean marginal bone loss.

All treated patients were periodontally healthy before they took part in the actual investigations. It is widely clinically accepted that, in periodontally compromised patients, implants are only placed after successful periodontal therapy. Already in the nineties, it has been proved that individuals with a strong susceptibility to periodontal diseases can be treated successfully with osseointegrated implants [42]. Moreover, Meyle et al. have recently demonstrated stable clinical and radiographic implant results in patients with a previous history of periodontitis [43]. On the other hand, it is known that patients with a history of periodontitis yield lower survival, significantly higher complications, and significantly lower success rates compared with patients who had lost their teeth for reasons other than periodontitis [44, 45]. Heitz-Mayfield and Huynh-Ba have shown in a review that the combination of a previous experience of periodontal disease and smoking increases the risk of implant failure and marginal bone loss around implants [46]. The present review reports on chronic, moderate, and advanced periodontitis. This implies that the compromising condition for all these periodontal subgroups is comparable. However, it is likely that, in this way, a bias is introduced. Indeed, Mengel and Flores-de-Jakoby have shown that patients treated for generalized aggressive periodontitis experience more attachment loss and bone loss when compared with patients treated for chronic periodontitis [47]. In addition, these patients are clearly more prone to late failure [47]. Similar findings have been reported by De Boever et al. who have shown that, unlike patients with chronic adult periodontitis, patients with generalized aggressive periodontitis exhibit more peri-implant pathology and marginal bone loss and display lower implant survival rates [48]. In accordance with the manufacturer’s instructions, all turned surface implants in this review were placed in the classic Bränemark two-stage submerged protocol, while the other implants were placed non-submerged. Recent studies have shown comparable outcomes for both treatment modalities [49, 50].

Bias is present in the included papers, and this can have a substantial impact on our findings. For example, in the studies by Wennström et al. and Nicu et al., smoking is a confounding factor, since both non-smokers and smokers have been combined [38, 40]. However, Cavalcanti et al. have performed a retrospective multicenter cohort study and have demonstrated almost twice as many implant failures in smokers compared with non-smokers [51]. Others have reported that smoking is a significant risk factor for early implant failures [52]. Subsequently, Sayardoust et al. and Nicu et al. have included fully edentulous patients in addition to partially edentulous patients [37, 40]. In the latter, the remaining dentition was able to serve as a source of pathogens, unlike the edentulous patients where prominent pathogens disappeared following dental extraction [20, 53, 54]. Another confounding factor could be the different sites of placement in the mouth. The loading of the implants differs between the anterior and posterior areas, and this could play a role in the measured outcomes [55]. Furthermore, bone quality is different between the maxilla and the mandible. Although implants have been installed arbitrarily in both jaws, more bone loss in the maxilla has been reported in clinical follow-up studies, without a real explanation for this phenomenon [56]. It is worth to note that all implants inserted in the reviewed papers have been placed in areas with good bone quality and under ideal conditions (e.g., implant placement predominantly in pristine cortical bone; Lekholm and Zarb type I and II bones). Indeed, under these conditions, one may expect a difficulty in detecting any differences due to the surface characteristics. Differences are more likely to be detected under conditions which are less ideal. The prescription of antibiotics is another potential confounding factor (Additional files 1, 2, 3, and 4). Two of the selected studies did not report on the intake of this prophylactic medicine [36, 39]. Keenan and Veitz-Keenan have recently suggested in their systematic review that a prophylactic antibiotic regimen reduces the failure of dental implants placed under ordinary conditions [57]. The osseointegrated implants have been provided with single unit crowns or fixed partial dentures. Only Nicu et al. have also included overdenture patients in their study [40]. Another important item that has not been addressed in any of the selected studies is the way in which the implant-supported restorations were connected to the fixture. In a recent systematic review, Lemos et al. have indicated that cement-retained, fixed implant-supported restorations show less marginal bone loss, fewer prosthetic complications, and higher implant survival rates compared with screw-retained, fixed implant-supported restorations [58]. However, this review should be interpreted with caution because of the few RCTs included in the analysis and the maximum observation time of 5 years of these meta-analyzed studies. The occurrence of peri-implantitis was reported in only one out of the six studies. Wennström et al. have reported a total of 4.7% peri-implantitis in the entire patient population [38]. The list of the six articles included five different surfaces. Machined implants are considered to be minimal rough [2]. In the moderately rough group, a significant heterogeneity has been observed: titanium plasma-sprayed, oxidized, titanium dioxide-blasted, and TiUnite-surfaces with different characteristics. The authors excluded the Donati et al. 20-year results of the Wennström et al. paper because of heterogeneity in follow-up time [59]. Further aspects that could contribute to bias are methodological factors. A risk of an overestimation exists due to analyzing the data on the implant level rather than the patient level, which is the result of the larger number of implants placed.

The heterogeneity and the variability in the study designs, together with the fact that most previous studies have not reported on confounding factors, make it difficult to draw definitive conclusions. In addition, the broad confidence intervals provide an uncertain outcome. In spite of their relatively higher failure rate, machined implants have possible advantages on the long term, because they attract less plaque once they become exposed after some years. Jungner et al. have shown equally high long-term survival rates, stable marginal and apical bone levels, and good peri-implant soft tissue health for turned and oxidized implants placed in grafted maxillary sinus floors [60]. Previous reviews by Quirynen et al. and Esposito et al. have shown a tendency towards more bone loss and higher incidence of implant loss around rougher surface implants [1, 34]. In an updated review on 27 randomized controlled trials in 1512 patients and 3230 implants, Esposito et al. have reported no evidence of a link between the type of dental implants and improved long-term success [61]. Simultaneously, they have stated that some limited evidence is present showing that implants with machined surface were less prone to bone loss related to peri-implantitis compared with implants with rougher surfaces. In a recent review, Doornewaard et al. have reported that peri-implant bone loss around machined implant systems was significantly lower than around moderately rough and rough implant systems [62]. However, they have concluded that the impact of surface roughness alone seems rather limited and of minimal clinical relevance.

## Conclusions

Due to lack of long-term data (> 5 years), the heterogeneity and variability in study designs and lack of reporting on confounding factors, definitive conclusions on differences in implant survival, and mean marginal bone loss between machined and moderate rough implants in periodontally compromised patients cannot be drawn. In order to understand whether or not machined surfaces are superior to moderately rough surfaces in patients with a history of periodontal disease, well-designed long-term randomized controlled trials are needed.

## Additional files

Additional file 1: Conflict of interest form (PDF 569 kb)

Additional file 2: Conflict of interest form (PDF 1224 kb)

Additional file 3: Conflict of interest form (PDF 569 kb)

Additional file 4: Conflict of interest form (PDF 569 kb)

## Abbreviations

AE: Acid-etched
Ant: Anterior
FPD: Fixed partial denture
Full: Fully edentulous
HA: Hydroxyl apatite
HS: Hybrid surface
Mand: Mandible
Max: Maxilla
MS: Machined surface
ND: No data
NR: Not reported
NS: Non-smoking
Partial: Partially edentulous
PC: Periodontally compromised patient
PH: Periodontally healthy patient
Post: Posterior
Pros: Prospective
Retro: Retrospective
RS: Rough surface
S: Smoking
SLA: Sandblasted acid-etched
TPS: Titanium plasma sprayed
